# Consumer segmentation based on three dimensions of sustainable food consumption: a simultaneous analysis of meat, organic food, and sweet snack purchases based on household panel data in Germany

**DOI:** 10.3389/fnut.2023.1140636

**Published:** 2023-06-27

**Authors:** Isabel Schäufele-Elbers, Meike Janssen

**Affiliations:** ^1^Faculty of Economics, Free University of Bozen-Bolzano, Bruneck, Italy; ^2^Department of Management, Society and Communication, Copenhagen Business School, Frederiksberg, Denmark

**Keywords:** sustainable food consumption, organic food, meat reduction, healthy diets, actual purchase data, household panel data

## Abstract

The literature on sustainable food consumption laments two major gaps: First, the majority of previous studies analyzed consumer behavior based on survey data on consumers’ self-reported behaviors and attitudes. Second, most existing studies focused on one dimension of sustainable food choices. This paper identifies and analyzes consumer segments based on the actual purchases of 8,400 households recorded in the GfK household panel data from Germany. We used three indicators of sustainable food consumption behavior: (1) the purchase of organic products as a proxy for the environmental impact of diets, (2) the purchase of meat as a proxy for the climate impact of diets, and (3) the purchase of sweet snacks as a proxy for the healthiness of a diet. The analysis yielded two larger segments with high expenditure shares for one type of unsustainable food (meat/sweet snacks, respectively), two small segments with above average (medium/high) expenditure shares for organic food, and a large ‘mainstream’ segment. The five consumer segments were further analyzed regarding the observed attitude-behavior gap, and the actual prices paid in different product categories. Clear gaps between stated and actual behavior were revealed with interesting differences between the five segments and the three sustainability characteristics. The analysis is a vital starting point for designing a holistic policy instrument mix to close the gaps and to reach a sustainable transformation of the food system.

## 1. Introduction

Current food production systems and dietary patterns prevalent in Western countries have significant negative impacts on the environment and human health and are therefore considered unsustainable. Sustainability problems arise along the entire value chain from primary agricultural production to food processing, transportation, and consumption. The production of food is very resource intensive and consumes a lot of land, water, and energy. The current food system in high-income countries contributes significantly to climate change, biodiversity loss, and diet-related diseases (1–3).

The necessary transition of food consumption patterns in high-income countries encompasses two elements (4). First, is the shift toward food produced with (more) sustainable methods, e.g., organic food. A second challenge is the transformation of dietary patterns away from animal and highly processed foods toward a whole food and (more) plant-based diet (5). The reduction of meat consumption is an all-encompassing objective to mitigate climate change and other externalities, but also to reduce health-related problems. In particular, the reduction of red and processed meat is associated with positive health and environmental effects (6). In addition to the reduction of meat, a healthy diet should reduce the intake of highly processed food (high in sugar, fat, or salt) and contain an unrestricted consumption of a variety of plant foods (e.g., legumes, whole grains, fruits, and vegetables) (5).

According to previous studies, there has been an increase in consumers’ preferences for sustainable and healthy diets in many Western European countries (7, 8). However, the market share of organic food is still at a low level, meat consumption is far too high and the consumption of food with high added sugar is predominant in the Western world. The discrepancy between consumers’ positive attitudes and purchase intentions and the low level of action (“attitude/intention-behavior gap”) is one of the key issues in consumer behavior research (9, 10). The reasons for the discrepancy are socially desirable responses that overestimate attitudes (11) on the one hand and contextual factors that prevent positive attitudes from transforming into behavior on the other hand (12).

The literature on sustainable food consumption laments two major gaps: First, the majority of previous studies segmented consumers based on survey data on consumers’ self-reported behaviors and attitudes. Real market data, i.e., food category specific expenditures and real prices paid, is scarcely examined because it is challenging for researchers to acquire such data and link it to data on consumers’ psychological constructs. However, relying on self-reported preferences and willingness-to-pay can have serious implications for the validity and reliability of segmentation studies and probably overestimate the segment of sustainable consumers. It is vital to analyze real purchase data and categorize to what extent food consumers can contribute to the transformation of the food system (8). Second, most existing studies focus on one dimension of sustainable food choices, e.g., organic food, local food, or fair-trade products (as prominent examples of food produced with more sustainable production methods), or plant-based food or healthy food choices (as prominent examples of more sustainable types of food).

Only a few segmentation studies have analyzed *several* types of sustainable food behaviors simultaneously (13). However, it is important to analyze sustainable food choices in a more holistic way to shed light on the preferences of different consumer segments to account for the complexity of the subject and the potential diversity among consumers. Some consumer segments may focus on the purchase of food from sustainable production systems (e.g., organic food), others may focus on dietary changes to behave more sustainably (e.g., eat less meat), some may do both, while yet others may not behave sustainably in any regard (13).

The aim of this paper is to identify and analyze consumer segments based on three dimensions of sustainable food choices: (1) the purchase of organic products as a proxy for the environmental impact of diets, (2) the purchase of meat as a proxy for the climate impact of diets, and (3) the purchase of sweet snacks as a proxy for the healthiness of a diet The consumer segmentation analysis presented in this paper is based on *actual* purchases of 8,400 households recorded in the GfK household panel data from Germany. The data further allows us to detect mutual relations between the three dimensions of sustainable food choices. The identified segments will be profiled and compared according to food values, purchase intentions, and socio-demographics. In addition to that, the available data allows for the analysis of real prices paid in different product categories. Knowledge of real consumer behavior in three dimensions of sustainable diets combined with real prices paid, psychological and sociodemographic data is a vital starting point for policymakers to design instruments to reach a sustainability transformation of the food system.

## 2. Segmentation studies in the field of sustainable food consumption

Dividing consumers into different homogeneous segments allows for the capture of heterogeneous consumer preferences and non-linear relationships, and gives the opportunity to develop targeted strategies to support the development of sustainable food systems (14). Not accounting for heterogeneity and just relying on mean preference coefficients runs the risk of overlooking (relatively small) consumer segments, especially in the case of opposing preference structures (e.g., preference for organic food versus aversion toward organic food) since differences can cancel out at the aggregated level (15).

Segmentation studies in the field of sustainable food consumption predominantly analyze consumer preferences for *one* specific sustainable product characteristic (e.g., organic production or local origin) or the degree to which a specific behavior, for example meat reduction, is carried out.

We found only two studies (7, 13) that focused on both types of sustainable behaviors, namely sustainable product choices *and* specific dietary behaviors. The study by Verain et al. (13) analyzed different sustainable product choices (organic, free-range, and products with a sustainability label) in different product groups (meat, dairy, and fruits and vegetables) and focused on two specific meat consumption behaviors (reducing meat portion size and reducing meat consumption frequency). The study by Brunin et al. (7) focused on organic food choices for 264 food and beverage items and considered healthy and plant-based diets.

*Actual* purchase data has hardly ever been used in segmentation studies. One exception is the study by Sarti et al. (8), which analyzed actual shopping behavior for sustainability *and* health-related product labels (social equity, ecological, health products, organic, and vegan); three consumer segments were identified with only one small segment of sustainable consumers (7%) that purchased relatively high numbers of products with health, environmental, and social benefits. However, the study only focused on product choices and not on sustainable dietary behaviors such as meat reduction. Moreover, the sample was limited to relatively few customers of one supermarket chain in Italy (UniCoop Tirreno) who were members of the voluntary loyalty card program (*n* = 132). The authors point to the fact that future research should study consumer purchases across larger samples to identify more differentiated consumer segments.

Two large-scale epidemiological studies used detailed information on types and amounts of food actually consumed. Brunin et al. (7) analyzed individuals’ dietary behavior as well as organic food consumption in France. Participants completed self-administered food frequency questionnaires in 2014 and 2018 that covered the consumption of 264 foods and beverages (*n* = 13,292 consumers). The segmentation study was based on a set of variables that reflected nutritional quality of the food consumed, as well as the consumption of plant-based food and organic food, to analyze sustainable consumption patterns between 2014 and 2018; six clusters were identified, referred to by the authors as ‘clusters of changes in consumption’ (7). Vieux et al. (16) analyzed national food consumption surveys in 5 European countries based on 48 h recalls and 3-, 4-, and 7-day dietary records (diaries; *n* = 8,302 consumers), and carried out a segmentation analysis based on dietary greenhouse gas emissions and nutritional quality resulting also in a six-cluster solution (16).

Most other segmentation studies were based on antecedents of real purchase behavior, i.e., the internal psychological processes of decision-making, which are necessarily related to real behavior but can only be used as proxies for the ‘real’ economic activity. Key proxies to explain and predict sustainable consumer behavior are foremost ‘values and concerns’ regarding personal health and the environment [see for example (17)], and ‘attitudes’ toward sustainable products [see for example (18)]. These processes in turn influence the formation of ‘intentions’ to purchase sustainable products or to change dietary behavior [see for example (19)].

The following sections give a brief overview of the results of previous segmentation studies that used antecedents of actual sustainable consumption behavior, i.e., values, attitudes, intentions and reported consumption behavior in order to allocate consumers to segments of sustainable consumption. To be considered for inclusion, studies had to conduct empirical analyzes of primary data, leading to the identification of consumer segments related to sustainable food consumption. The following section is structured according to the three most frequently identified consumer segments (14): ‘sustainable consumers’, ‘non-sustainable consumers’, and segments that lie ‘in-between’ these two distinct groups of consumers.

### 2.1. Sustainable consumers

The segment of ‘sustainable food consumers’ is often described as being involved in sustainability issues, environmentally concerned, and more likely to buy organic or animal welfare products [see the literature review of (14)]. The size of the sustainable food consumer segment has been explored in various survey studies that used stated preferences, concerns, values and/or attitude, and it has been found that it typically ranges from around 30 to 40% of the total sample. This segment was identified in a Dutch study focused on food consumption in general (13), as well as in an Italian study on canned tuna fish (20). Additionally, a cross-national study on wine consumers (21), an Italian wine consumer study (22) and a German wine study (23) also reported that a similar proportion of the population belonged to this segment.

Fewer studies considered sustainable consumption more holistically and addressed health aspects in addition to environmental ones. This stream of research provides evidence that a substantial segment of sustainability oriented wine consumers in the United States, the United Kingdom, and Germany (17), as well as food consumers in Poland and the Czech Republic (24), in Hungary (25), in four EU countries (United Kingdom, Germany, Belgium, and the Netherlands; 26) and in the Netherlands (27) prioritize environmental and health factors when making purchasing decisions. These studies suggest that a considerable share of consumers holds preferences for environmentally-friendly *and* healthy diets (28, 29). The segment of ‘committed organic consumers’, for example, is predominantly motivated by the desire for healthy and natural food as well as concern for the environment (30, 31). This observation was also confirmed by the two large-scale epidemiological studies that used detailed information on foods actually consumed, i.e., reported consumption behaviors. Brunin et al. (7) found a segment of French consumers that had already initiated a transition toward sustainable diets (16%): high levels of organic products, healthier food choices, more plant-based foods. Similar findings were observed by the cross-European study of Vieux et al. (16): the segment the authors referred to as‚ more sustainable’ (18%) was characterized by high levels of plant-based food consumption and healthy dietary patterns (slightly higher intake of dairy products, lower intake of meats, and lower intake of sugar/confectionaries, soft drinks, and alcoholic beverages), which the authors referred to as the ‘best compromise’ between nutritional quality and dietary greenhouse gas emissions.

### 2.2. Non-sustainable consumers

Studies based on stated preferences, values, and/or attitudes have consistently revealed that the proportion of ‘non-sustainable consumers’, those who do not prioritize sustainability in their purchasing decisions, is small across a range of food products and countries. Specifically, research on milk, yogurt, and apples in Germany (32), canned tuna fish in Italy (20), wine in Germany (23), and a Romanian study on food consumption in general (33) all found that this segment was around 10% in size. Consumers who belong to this segment hold rather negative attitudes toward sustainably produced products and are often very price-conscious.

In the two epidemiological studies, the share of this consumer group was somewhat higher Brunin et al. (7) identified a segment of 16% with a low level of organic food consumption in 2014 that further decreased in 2018. Moreover, this segment revealed changes toward an unhealthy diet (increase in consumption of unhealthy plant products, animal products and alcohol). Interestingly, the study of Vieux et al. (16) revealed two clusters with different types of unsustainable behaviors. The “Highest greenhouse gas emissions” segment (14%) was characterized by high consumption of meat, animal fats and alcoholic beverages and low consumption of meat substitutes, vegetable fats, and plant-based composite dishes. The “Lowest greenhouse gas emissions” segment (24%) showed unhealthy consumption patterns characterized by the highest intake of soft drinks, sugar/confectionaries, and snacks/desserts.

### 2.3. Consumers ‘in-between’

The segment that lies in-between these two rather distinct clusters was found to be by far the largest segment (around 40 to 70% of the sample) in the majority of studies This consumer group is very heterogeneous and previous studies have given them very different names, for example ‘potential consumers’ to characterize its diverse nature. Some studies even identified more than just one segment in-between the ‘sustainable consumers’ and the ‘non-sustainable consumers’ (14). Due to this heterogeneity, this cluster will be described in more detail here.

Several studies refer to individuals in this segment as ‘average consumers’ because the variables related to sustainable behaviors are very close to the sample means (34) and less distinct (32). This implies that consumers are rather indifferent and/or unaware regarding different sustainability issues. Other food attributes such as price (14, 35), health (14) or country of origin (37) seem to be more important to the consumers of this segment. Some studies describe this cluster as ‘inconsistent’ regarding certain values and behaviors. In the studies by Forleo et al. (20) and Gazdecki et al. (18), consumers in this segment generally hold rather positive attitudes toward sustainable consumption but they do not (yet) translate their positive predisposition into sustainable behaviors. In the context of organic product choices, high prices and low availability are often given as reasons for this attitude-behavior gap; the corresponding segment is named ‘occasional organic buyers’ in this stream of literature (36). Health motives are the predominant purchase driver for ‘occasional organic buyers’; environmental and ethical motives are less relevant (37).

Studies that analyzed different dimensions or types of sustainable consumption (additionally) identified segments that performed well with regard to one sustainability dimension/type, but not with regard to others. Some studies identified a segment of consumers only attentive to health aspects (not interested in environmental issues) which was described as ‘egoistic’ to highlight the contrast to the cluster motivated by ‘altruistic’ values such as environmental attributes (for example 38). The study of Verain et al. (13) identified one segment with a strong preference for the purchase of sustainably produced products (but showed relatively low performance of meat reduction) and one segment that reduced meat consumption (but had a low share of sustainable product choices).

Similar results were observed in the epidemiological studies of Brunin et al. (7), and Vieux et al. (16). Brunin et al. (7) found three consumer segments (‘cluster toward healthy food’ (13%), ‘cluster toward plant food’ (23%) ‘cluster toward healthy plant food’ (7%)’) that improved at least one sustainability dimension in their diets over the period 2014 to 2018 (but had not yet achieved sustainable dietary transition). Vieux et al. (16) found a huge segment of consumers (33%) with intermediate dietary greenhouse gas emissions and nutritional quality and, moreover, a rather small segment (10%) with high dietary greenhouse gas emissions and contradictory values of dietary quality indicators (high in beneficial nutrients, but also high in sodium, free sugars, and saturated fatty acids).

In summary, the results show that the segment of ‘non-sustainable consumers’ is rather small compared to the segment of ‘sustainable consumer’. However, epidemiological studies show slightly higher shares for the segment of ‘non-sustainable consumers’ and one study even found two clusters with different types of unsustainable behaviors. Moreover, the results highlight the relation between environmental and health concerns as potential drivers of organic food purchase behavior and reduced meat consumption and that both dimensions are linked to either environmental concerns, or health concerns, or both under a ‘good for me good for the planet’ concept. Furthermore, the segment between sustainable and non-sustainable consumers is by far the largest segment and very heterogeneous. This group is often referred to as ‘average consumers’ with less distinct sustainable behaviors, other food attributes such as price and health are more important to them. Overall, these studies highlight the need and the potential for more targeted interventions to promote sustainable behaviors among this consumer group.

## 3. Materials and methods

### 3.1. GfK household panel data

Household panel data are most appropriate to measure real purchase behavior and analyze attitude-behavior relations, but they are expensive and difficult to access for researchers. Surveys and purchase experiments are more commonly used, despite their limitations in measuring behavior accurately. Surveys rely on self-reporting, which can be subjective and influenced by social desirability, while purchase experiments are often hypothetical and not incentive-compatible. Thus, the notable strength of household panel data is its relatively high degree of validity, which is deemed to be a critical advantage.

This research study is based on two panels of consumers from the GfK market research institute: ConsumerScan, which includes purchases of pre-packaged foods, and ConsumerScan FreshFood, which covers purchases of unpackaged foods. The sample for this study is comprised of 8,400 households in Germany who participated in both panels throughout 2016. The dataset includes information on total food purchases at the household level, including details on organic and conventional purchases. Additionally, the dataset includes information on purchases in various food categories such as meat and sweets. Throughout 2016, the households that participated in the study utilized an electronic device called the ElectronicDiary to register their food purchases. The device scanned the European Article Number (EAN) code, and additional details like price and store name were entered via the scanner’s keypad. In cases where the food items were not packaged and did not have an EAN code, like fresh produce, a code book was supplied. Additionally, every year, the head of each household was required to complete a written questionnaire containing more than 120 survey items that covered topics such as consumer lifestyle, values and attitudes toward food, and socio-demographic characteristics. The purchase data and survey questions were interconnected through a unique identification number, allowing for the linkage of purchases with food values in the database.

### 3.2. Indicators of sustainable food consumption behavior

The study considers three indicators to cover three dimensions of sustainability, i.e., environmentally-friendly farming practices, climate-friendly food choices, and healthy food choices. While this approach oversimplifies the complex issue of healthy diets from sustainable food systems (39–42), it is a very useful starting point from a methodological perspective to gain insights into food consumption and its multidimensional nature.

Household-level expenditure share aggregated on an annual level in 2016 was used to calculate the three indicators. Using expenditure share as a measure is preferable because it takes into account the relative importance of different food categories within the household’s overall budget. This can provide a more accurate reflection of the household’s priorities and preferences when it comes to food consumption. Moreover, using expenditure share adjusts for differences in household size and allows for comparisons across households with different budgets. The calculation of the indicators was performed as follows:

- expenditure share for organic food: expenditures for organic food (in €) in relation to the total expenditures for food (in €) as a proxy for environmentally-friendly food choices.
- expenditure share for meat: expenditures for meat (in €) in relation to the total expenditures for food (in €) as a proxy for the climate impact of diets, and
- expenditure share for sweet snacks: expenditures for sweet snacks (in €) in relation to the total expenditures for food (in €) as a proxy for the healthiness of a diet

Expenditures for meat comprise purchases of fresh pork, beef, and poultry, including both organic and conventional options. The food category of sweet snacks includes sweets, chocolates, and sweet bakery products, also in both organic and conventional varieties. Organic food includes organic products in all food categories (unpackaged and packaged food).

### 3.3. Cluster analysis

Cluster analysis was preferred over regression analysis in this study, as it allowed for the examination of the multi-dimensional nature of the phenomenon under investigation, involving three explanatory variables related to sustainable consumption. The cluster analysis was conducted with the R function for k-means clustering to identify homogeneous groups regarding sustainable purchase behavior. This method is most appropriate for huge samples as in our case. While latent class models are an option for conducting segmentation analysis, we opted to utilize K-means cluster analysis due to its ability to facilitate post-hoc examination of the clusters in terms of profiling variables, such as indicators of sustainable food consumption.

The k-means algorithm used was the Euclidean distance metric; target criterion was the minimization of variance within the clusters. A critical point is that the cluster assignment depends on the choice of the starting positions. Therefore, the analysis was carried out 2,000 times with random start values and the solution which minimized the error sum of squares to the largest extent was chosen (43).

The k-means cluster algorithm is very sensitive to extreme values, which is why we conducted a hierarchical cluster analysis (method: single linkage) to identify outliers. Two cases were identified as outliers and excluded from the cluster analysis.

For determining the optimal number of clusters, the k-means algorithm was run for five different numbers of clusters (from 3 to 7 clusters). The five solutions were then evaluated and compared based on three criteria (elbow method/within-sum-of-squares, silhouette score, and gap-statistic) as suggested by Malik and Tuckfield (44). A minimum of three clusters was chosen due to content-related considerations and previous knowledge; a maximum of seven clusters was chosen due to the considerations of manageability from a marketing perspective. First, the elbow method was used, which strives to find a compromise between the minimization of the within sum of squares (WSS) and the manageability of high cluster numbers. The error sum of squares declines with an increase in the cluster number, but the rate of decline might drop at some point, creating the ‘elbow’ shape and hinting toward the optimal number of clusters. Second, the gap statistic was examined (45), which compares the WSS value of the observed dataset to a dataset with no cluster structures (random distribution) and chooses the cluster number with the maximum value of the gap statistics (44). Finally, the silhouette coefficient was examined, which measures the similarity of each data point to its own cluster compared to other clusters (46). To confirm a real existing cluster structure, the average silhouette coefficient should be larger than 0.25, and preferably larger than 0.5 (46).

Finally, the clusters were analyzed for statistically significant differences in food values, purchase intentions, socio-demographics, and purchase behavior with the method of one-way analysis of variance (ANOVA) and pairwise comparisons of column proportions (z-test).

As a pre-step, scales for 10 food-related values were created based on 55 items included in the GfK annual survey of panel households. Cronbach’s alpha was used as a measure for internal scale reliability; all food-related value scales had Cronbach’s alpha values larger than 0.7. Appendix Table 1: Food-values scales.

## 4. Results

### 4.1. Description of the sample

Table 1 displays the socio-demographic features of the sample used in the study, which are compared with those of the general German population. However, it is not easy to make a direct comparison as the federal statistical office uses distinct age and income categories not directly comparable with the categories of the GfK consumer survey. Moreover, the education categories of the GfK survey incorporate both school-leaving and vocational qualifications. This makes it challenging to compare with the two separate statistics provided by the German federal office.

**Table 1:** Socio-demographic characteristics of the sample and the German population.

Socio-demographics (*N* = 8,400)	Sample %	Population %	
	Age of the head of household	Age of German residents older than 18 years^1^	
Up to 29 years	1.9	17.0	
30–39 years	10.1	14.2	
40–49 years	17.2	19.9	
50–59 years	24.8	50 up to under 75 years 37.8	
60–69 years	23.3		
70 years and older	22.6	75 years and older 11.2	
	Formal education of the diary keeper (including vocational school and university)	School-leaving qualification of German residents^2^	Vocational qualification of German residents^3^
Secondary general school	22.5	29.6	–
Intermediate secondary school	32.9	29.9	–
Qualified dual vocational training program	–	–	47.5
Special upper secondary school (vocational school)	8.0	–	8.8
University entrance diploma	14.1	32.5	–
University	22.5	–	18.0
Others	–	8	25.7
	Household net income	Net income of private households in Germany^4^	
Up to 749 Euro	3.5	Under 1,500 26%	
750–1,249 Euro	12.9		
1,250–1749 Euro	16.2	1,500-3,200 43%	
1750–2,249 Euro	18.8		
2,250–2,749 Euro	15.6		
2,750–3,249 Euro	12.8		
3,250–3,749 Euro	7.7	Over 3,200 31%	
3,750–4,999 Euro	9.2		
5,000 Euro and more	3.3		

^1^German Federal Statistical Office (47), table 12, 111–0004.

^2^German Federal Statistical Office (48), p. 88.

^3^German Federal Statistical Office (48), p. 90.

^4^German Federal Statistical Office (47), table 12, 111–0004.

Concerning age, the sample seems to be lacking in representation of young households, particularly those in the youngest age group, with only 2% of the sample compared to 17% in the overall population. In a third of the households, the person responsible for the purchase diary had a university-entrance diploma or a university degree, which aligns with the distribution of the highest school-leaving qualification of the German population. However, it appears that high-income households were not adequately represented in the sample.

Overall, the findings indicate that the sample utilized in the study may not accurately reflect the characteristics of the German population. Therefore, it is important to exercise caution while generalizing the results to the overall population.

### 4.2. Descriptive analysis

The average expenditure share for organic food was 3.9% (mean = 0.039, SD = 0.081), with a highly skewed distribution. Half of the consumers spent less than 1.2% of their food expenditures on organic products (one quarter less than 0.04%) and only one quarter spent more than 3.5%, which is quite low compared to the high share of consumers (39%) who stated they preferred organic food when purchasing groceries upon being asked this question in the survey (Table 2).

**Table 2:** Indicators of sustainable food consumption–whole sample and by segment in %.

		Expenditure share organic	Expenditure share meat	Expenditure share sweet snacks
Overall sample (*N* = 8,398)				
Mean value		3.91	5.56	6.63
SD		8.07	3.92	4.17
Quartiles	25	0.44	2.73	3.73
	50	1.23	4.99	5.84
	75	3.55	7.64	8.64
Heavy organic buyers (*N* = 132)				
Mean value		51.28^a^	3.68^bde^	4.14^a^
SD		14.68	4.05	2.87
Quartiles	25	39.83	0.00	1.98
	50	45.50	2.56	3.75
	75	59.97	5.64	5.51
Medium organic buyers (*N* = 635)				
Mean value		18.15^b^	4.41^b^	5.24^ce^
SD		6.31	3.86	3.33
Quartiles	25	13.00	1.62	2.80
	50	16.57	3.69	4.58
	75	21.87	6.25	6.76
Heavy meat buyers (*N* = 2,067)				
Mean value		1.58^c^	10.55^c^	5.17^ce^
SD		1.99	3.21	2.59
Quartiles	25	0.30	8.28	3.24
	50	0.81	9.62	5.02
	75	2.03	11.91	6.92
Heavy sweet buyers (*N* = 1,691)				
Mean value		1.64^c^	3.84^d^	12.78^d^
SD		2.01	2.67	3.96
Quartiles	25	0.34	1.66	10.14
	50	0.86	3.61	11.63
	75	2.16	5.64	14.12
Mainstream (*N* = 3,837)				
Mean value		2.19^d^	3.90^de^	5.04^e^
SD		2.35	1.99	2.14
Quartiles	25	0.49	2.39	3.41
	50	1.27	4.02	5.08
	75	3.09	5.57	6.76

^a,b,c,d,e^Mean values of segments with different letters differ significantly (*p* < 0.05).

The average expenditure share for fresh meat was 5.6% (mean = 0.056, SD = 0.039). On average, pork accounted for half of the expenditures, beef for a quarter, and poultry for another quarter. A quarter of the consumers spent more than 7.6% of their food budget on meat.

Regarding sweet snacks, the average expenditure share was 6.6% (mean = 0.066, SD = 0.042). A quarter of the consumers spent more than 8.6% of their food budget on sweet snacks.

The relationships between the three sustainability dimensions are significant but very weak. The expenditure share for organic food is negatively correlated with the expenditure share for sweet snacks (*r* = −0.134) and the expenditure share for meat (*r* = −0.114). However, the expenditure shares for meat and sweet snacks are also negatively correlated (*r* = −0.193), suggesting that sustainable consumption behavior in one dimension of sustainability does not necessarily go hand in hand with sustainable consumption behavior in other dimensions. This leads to the proposition that a unidimensional and linear data analysis approach is not sufficient to capture the complexity, and highlights the need for cluster analysis to analyze sustainable food choices in a more holistic way so as to account for the potential diversity among consumers.

### 4.3. Cluster analysis

**Figure 1:**
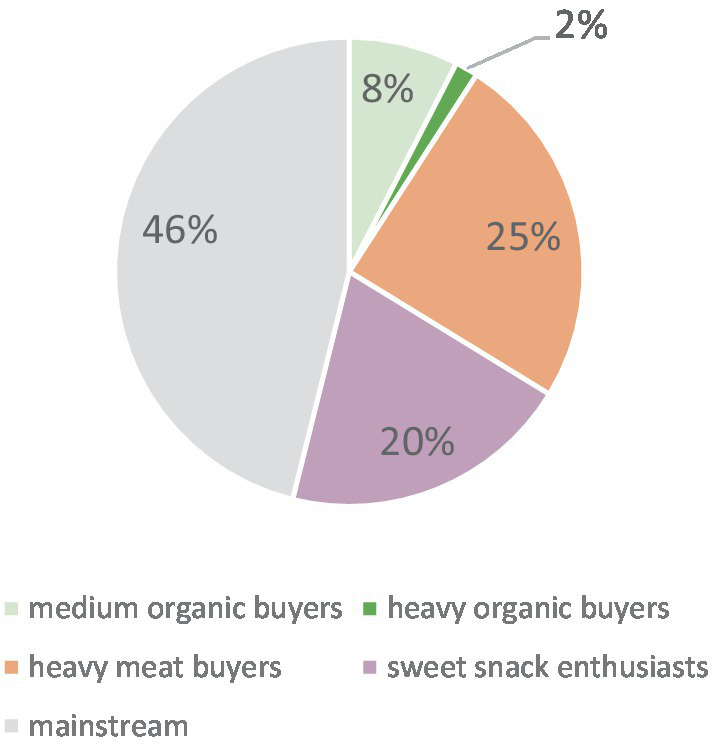
Share/size of the segments.

**Figure 2:**
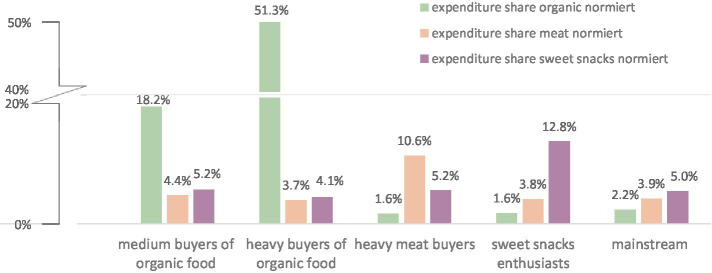
Cluster centers of the k-means cluster analysis.

The graphical analysis of the elbow criterion as well as the gap statistic suggested choosing the 5-cluster solution. The silhouette coefficient achieved the highest value for the 3-cluster solution but still confirmed an acceptable cluster structure for the 5-cluster solution with an average silhouette width of around 0.3. We therefore chose the 5-cluster solution. It is important to note that the non-inclusion of processed meat in the meat indicator due to data unavailability, might have led to biased results. Hence, it is advisable to approach the following findings with caution. Figure 1 shows the relative size of the five consumer segments identified; Figure 2 displays the cluster centers of the k-means cluster analysis. The analysis yielded two larger segments with high expenditure shares for one type of unsustainable food (meat/sweet snacks, respectively), two small segments with above average (medium/high) expenditure shares for organic food, and a large ‘mainstream’ segment:

– Heavy meat buyers: The second largest cluster (25% of all households) spent a high share of expenditure on meat (11%). Their expenditure share for organic food was below average (1.6%), sweet snacks purchases were on an average level.
– Sweet snacks enthusiasts: The third largest consumer segment (20% of all households) purchased sweet snacks extensively (13% of all food expenditures). The expenditure share for organic food was below average and similar to the heavy meat buyers (1.6%). The expenditure share for meat was on an average level.
– Medium organic buyers: This smaller segment (8% of all households) spent 18.2% of their expenditure on organic food. Their expenditure shares for meat and sweet snacks were on an average level.
– Heavy organic buyers: The smallest consumer segment (2% of all households) had a very high expenditure share for organic food. On average, they purchased every second item in organic quality (expenditure share of 51.3%). Sweet snack consumption was significantly lower compared to all other clusters (expenditure share: 4.1%). Their expenditure share for meat was slightly below average (expenditure share: 3.7%).
– Mainstream consumers: The largest segment (46% of all households) had relatively low expenditure shares for organic food (2.2%), below average expenditure shares for meat (3.9%), and average expenditure shares for ‘sweet snacks’ (5.0%).

### 4.4. Food values, purchase intentions, dietary behavior, and socio-demographics

In the following sections, the segments are profiled according to food values (Table 3), purchase behavior (Tables 4, 5), intentions (Table 6), and socio-demographics (Appendix Table 2).

**Table 3:** Psychographic profiles of the segments–food values.

Food values	Medium buyers of organic food	Heavy buyers of organic food	Heavy meat buyers	Sweet snacks enthusiasts	Mainstream
Body consciousness	2.83^c^	2.70^abc^	2.71^ab^	2.66^b^	2.77^ac^
Convenience food	2.11^a^	1.88^c^	2.51^b^	2.76^e^	2.60^d^
Environmental protection	3.76^a^	4.03^c^	3.24^b^	3.24^b^	3.30^d^
Fair trade	3.80^a^	4.18^c^	2.89^b^	2.96^be^	3.00^de^
Fast food	1.65^ab^	1.66^ab^	1.58^b^	1.85^c^	1.72^a^
Avoiding health risks	2.59^a^	2.55^a^	2.56^a^	2.59^a^	2.60^a^
Local food	3.97^a^	4.04^a^	3.51^bc^	3.45^c^	3.53^b^
Natural food	3.81^a^	4.18^c^	3.10^b^	3.10^b^	3.14^b^
Quality and enjoyment	3.33^a^	3.38^ab^	3.23^b^	2.98^d^	3.17^c^
Simple and easy cooking	3.17^a^	3.08^ac^	3.03^c^	3.44^b^	3.23^a^

^a,b,c,d,e^Values of segments with different letters differ significantly (*p* < 0.05).

**Table 4:** Average price paid by different consumer segments (in €).

Price in €/Kg	Medium organic buyers	Heavy organic buyers	Heavy meat buyers	Sweets snacks enthusiasts	Mainstream	Overall
Fresh beef	8.49^a^	7.76^ac^	8.61^a^	5.44^b^	6.71^c^	7.07
Fresh pork	6.53^a^	5.83^abcd^	5.85^b^	5.03^c^	5.55^d^	5.60
Fresh poultry	7.09^a^	7.50^ab^	5.85^b^	5.40^c^	5.64^c^	5.78
Sweet snacks	10.13^a^	12.88^b^	7.92^c^	7.49^d^	8.56^e^	8.37

^a,b,c,d,e^Values of segments with different letters differ significantly (*p* < 0.05).

**Table 5:** Average purchases in kg/l per household in different product groups for consumer segments.

	Medium organic buyers	Heavy organic buyers	Heavy meat buyers	Sweets snacks enthusiasts	Mainstream	Whole sample
Alcoholic beverages	64	49	108	47	106	91
Ready to eat desserts	4	3	7	10	7	7
Sweet and salty snacks (including nuts and seeds)	26	21	28	51	25	31
Fish	8	6	9	6	8	8
Poultry	6	4	13	5	5	7
Beef	4	3	8	2	3	4
Pork	8	6	27	10	10	14
Poultry products	2	1	3	2	2	2
Cheese	16	17	15	14	16	16
Frozen food	22	15	33	32	30	30

**Table 6:** Share of agreement (in %) with statements on intentions to follow a sustainable diet.

	Medium organic buyers	Heavy organic buyers	Heavy meat buyers	Heavy sweet buyers	Mainstream	Overall
‘We intentionally reduce our meat consumption’						
I fully disagree	8.7^a^	4.8^a^	23.5^b^	18.2^c^	17.9^c^	18.4
I rather disagree	11.2^a^	5.6^a^	24.3^b^	22.0^b^	19.2^c^	20.2
Neither nor	17.1^a^	13.6^a^	24.0^b^	23.1^b^	23.4^b^	22.9
I rather agree	31.6^a^	31.2^a,c^	22.5^b^	25.7^c^	27.1^c^	26.1
I fully agree	31.4^a^	44.8^c^	5.7^b^	11.0^d^	12.4^d^	12.5
‘When I buy food, I prefer organic products’						
I fully disagree	3.1a	1.5a	33.6b	31.6b	28.1c	18.4
I rather disagree	9.3a	2.3c	29.3b	28.3b	28.1b	20.2
Neither nor	14.6a	4.6c	21.9b	24.4b,d	24.7d	22.9
I rather agree	44.1a	21.5c	13.1b	13.1b	16.2c	26.1
I fully agree	28.9a	70.0c	2.1b	2.6b	2.9b	12.5
‘In my diet, I avoid everything that is harmful to health’						
I fully disagree	7.2^a,b,c^	3.8^b^	9.4^c^	9.6^a,c^	8.9^a,c^	18.4
I rather disagree	16.2^a^	15.4^a,b^	22.5^b^	21.9^b^	21.7^b^	20.2
Neither nor	27.8^a^	20.0^a^	34.2^b,c^	35.1^b^	32.3^c^	22.9
I rather agree	35.7^a^	46.9^c^	24.9^b^	25.0^b^	27.9^d^	26.1
I fully agree	13.0^a^	13.8^a,c^	9.0^b,c^	8.4^b^	9.2^b,c^	12.5

^a,b,c,d^Shares of segments with different letters differ significantly (*p* < 0.05).

#### 4.4.1. Medium and heavy buyers of organic food

The two segments of ‘medium / heavy organic food buyers’ (together 10% of the sample) had high preferences for local, natural, and fair-trade food and placed great importance on environmental protection, significantly higher than the three other segments (Table 3). Heavy organic buyers placed slightly higher importance on the above food values than medium buyers (differences statistically significant except for local food). Both segments had very low preferences for convenience food and for ‘simple and easy cooking’. Moreover, ‘medium and heavy organic food buyers’ paid high mean prices for sweet snacks (Table 5), which indicates they preferred high-quality products.

Despite these similarities between the two organic segments, we found that the ‘heavy organic buyers’ purchased significant lower quantities of sweet and salty snacks (Table 4) as well as meat compared to all other clusters (Table 2; ‘medium organic buyers’ were in the middle range). Even though two thirds of ‘the medium organic buyers’ had the intention to reduce their meat consumption (Table 6), they bought relatively high amounts of meat, even at the same level as the ‘mainstream’ segment (Table 4). However, differences between the types of meat were found: medium organic buyers consumed less pork but the second highest amount of beef and poultry (only the ‘heavy meat buyers’ purchased more) (Table 4).

A high share of people in the two organic segments belonged to the highest income group and held a university degree. Heavy organic buyers were more likely to be in the youngest age groups (under 40 years old).

#### 4.4.2. Heavy meat buyers

The segment ‘heavy meat buyers’ (25%) showed low preferences for ‘simple and easy cooking’ and ‘fast food’ and high preferences for ‘quality and enjoyment’ compared to the segments ‘sweet snacks enthusiasts’ and ‘mainstream consumers’. The awareness of environmental issues and the preference for fair-trade food was low (Table 3). ‘Heavy meat buyers’ purchased the highest amounts of beef, pork, meat, fish, and poultry products and had a relatively high consumption of alcoholic beverages (Table 4). For beef, they paid the highest average price of all segments (but no statistically significant difference to the medium and organic buyers (Table 5). Approximately a third of the consumers in this segment reported intentionally reducing their meat consumption; half of the segment stated they did not do so. Only a small share of heavy meat buyers was younger than 40 years old; most were aged 50–69 years old. Income and formal education of the segment were relatively low.

#### 4.4.3. Sweet snacks enthusiasts

Sweet snacks enthusiasts (20%) had the highest preferences for ‘convenience food’, ‘fast food’ and ‘simple and easy cooking’ of all segments. Moreover, the segment attached low importance to ‘quality and enjoyment’ and ‘environmental protection’. This segment paid the lowest mean prices in all food categories (Table 3). In line with their high consumption of sweet snacks, they purchased the highest amount of ready to eat desserts. Alcohol consumption was, however, low (Table 5). Moreover, they purchased the lowest quantities of cheese. Income and formal education of the segment were relatively low, but did not differ significantly from the ‘heavy meat buyers’. People in this segment were more likely to be 40–49 years old.

#### 4.4.4. Mainstream

The segment of ‘mainstream consumers (46%)’ shared many similar food values with the heavy meat buyers’ and the ‘sweet snacks enthusiasts’; only that the mainstream consumers were slightly more environmentally oriented than the two latter segments. Moreover, the mainstream consumers were a little more body-conscious and quality-oriented than the ‘sweet snacks enthusiasts’, and slightly more convenience and fast-food oriented than the ‘heavy meat buyers’. Mainstream consumers purchased relatively high quantities of alcoholic beverages. People above 70 years of age were overrepresented in this segment. Formal education and income were in the middle range.

## 5. Discussion and conclusions

The main contribution of this study is the use of data on real purchase behavior and the simultaneous inclusion of behavior regarding food produced with more sustainable production methods (organic food) as well as dietary food choices concerning the types of food consumed (meat consumption and healthy eating). This made it possible to identify consumer segments with different *levels* and *types* of (un)sustainable consumption behavior and to analyze the gap between behavioral intentions and real purchase behavior.

Overall, consumer segments with more positive attitudes and intentions regarding sustainability showed more positive actual sustainable purchase behaviors. Thus, the results confirm the study of Brunin et al. (7) that food motives are useful predictors of sustainable consumption behavior. However, food values and intentions did not completely transmit into actual behaviors. Gaps between stated and actual behavior were revealed, and these gaps differed between the consumer segments and the different sustainability characteristics. The gap was highest for the segment of ‘heavy sweets buyers’ and ‘heavy meat buyers’: even though they showed rather unhealthy consumption patterns, one third of these segments agreed to avoid everything in their diet that is harmful to their health. Moreover, a considerable amount of ‘heavy meat consumers’ (27%) stated to consciously reduce meat consumption. Compared to statements on health and meat reduction, the attitude-behavior gap for the purchase of organic food seems to be relatively low. This finding is in line with several studies that confirm a relatively strong attitude-behavior relationship for organic food (49).

Only a small part of the population shows relatively sustainable consumption behaviors in all consumption dimensions considered (high consumption of organic food, low consumption of meat, sweet snacks, alcohol, processed foods) and is composed of younger people with higher education, which is in line with a recent large-scale epidemiologic study by Brunin et al. (7). However, even this relatively sustainable segment consumes a considerable amount of beef, 50% of it coming from organic livestock. Even though this alternative farming method is probably associated with positive effects for biodiversity compared with conventional beef production, organic meat production does not have particular advantages regarding climate impact (50). Moreover, cheese consumption (a product with high greenhouse gas emissions), is also relatively high in this segment. This result is similar to the study of Brunin et al. (7) who found that the segment with the lowest meat and processed meat consumption showed the highest quantity of dairy products.

The meat consumption quantities of medium organic buyers show that buying organic food does not necessarily go hand in hand with low meat consumption. While pork consumption in this consumer segment is on an average level, they consume relatively high amounts of beef and poultry. However, it is noteworthy that two-thirds of medium organic buyers state that they consciously reduce meat, i.e., they claim to already limit their consumption. Nevertheless, it cannot be clearly determined whether there is a gap between intention and behavior because the study did not examine the purchase of processed meat. It is possible that the medium organic buyers may have already reduced the consumption of processed meat, given their high environmental values and their low preference for processed food.

Almost half of the households clearly behave in a non-sustainable way, either due to high meat consumption (‘heavy meat buyers’, 20%) or due to high consumption of sweet snacks (‘sweet snack enthusiasts’, 25%). This proportion is significantly higher than in past studies based on self-reported values and intentions, where the proportion of non-sustainable consumers was around 10%. However, our results are similar to those by Vieux et al. (16) who found two non-sustainable segments, one with high greenhouse gas emissions (14% of the sample) and one with unhealthy dietary patterns (24% of the sample). Looking at the choice of organic-labeled food, our study reveals that 90% of consumers do not purchase organic food to a considerable extent, which is comparable to the study of Sarti et al. (8) which is based on actual purchase data as well. The authors found a segment of 71% which was not interested in the purchase of sustainability labels.

The present study identified several consumer groups that differ in their unsustainable purchase behaviors, which has important implications:

Interestingly, the ‘sweet snacks enthusiasts’ (20%) segment which attaches low importance to ‘environmental protection’ behaves sustainably with regard to the low climate impact of their diet. They do not attach a strong importance to environmental protection and the intention to reduce meat is average. Nevertheless, they have a low consumption of beef and cheese, which is positive for the greenhouse gas footprint. In addition, the diet of these consumers is composed of high proportions of sweets and processed foods, products with rather low greenhouse gas emission, however, with a high energy density and few nutrients (16). This segment has many similarities with the ‘lowest greenhouse gas emissions’ segment (24%) in the study of Vieux et al. (16): high intake of sugar/confectionaries and snacks/desserts and low meat and dairy consumption. The rather unhealthy eating behavior of the ‘sweet snack enthusiasts’ goes hand in hand with a preference for convenience and low-priced food, which is comparable to the findings of Brunin et al. (7).

The second segment of concern in terms of sustainability is that of ‘heavy meat buyers’ (20%) with a high consumption of all types of meat. The diet of this segment causes high amounts of greenhouse gas emissions, which is also reflected in the low importance these consumers attach to environmental protection and other sustainability aspects. This segment is comparable to the segment of ‘highest greenhouse gas emissions’ in the study of Vieux et al. (16), both in size, and high meat and alcohol consumption. Surprisingly, a large proportion of ‘heavy meat buyers’ (28%) in this study state they are consciously eating less meat. Moreover, the ‘heavy meat buyers’ attach importance to high-quality food. These results are in line with the study by Bakker and Dagevos (51) who found that a quarter of the Dutch population are so-called ‘heavy meat buyers’. They suggest that the image of meat as healthy and the culture and traditions surrounding the preparation and consumption of meat, especially the association of superiority, are responsible for the gap between attitudes and behavior.

The present study suggests that many consumers behave sustainably in only *one* dimension of sustainability (either climate-friendly, healthy, or environmentally-friendly) while they follow rather unsustainable dietary patterns in other dimensions. Most policy instruments for fostering sustainable consumption are ‘one-dimensional’ by design, i.e., they focus on *one* specific dimension of sustainability, e.g., health aspects *or* organic production (52). The introduction of climate labels or climate taxes are currently discussed with great controversy (53–56). One of the paradoxes with one-dimensional sustainability measures is the danger of ‘licensing effects’. For example, ‘heavy sweet snack consumers’ could use a widely introduced climate label to justify high sweet snack consumption. In this case, the climate label would thus act as a ‘license’ for unhealthy eating.

We therefore recommend considering interactions with other sustainability dimensions before introducing ‘one-dimensional’ policy measures. A positive example of a multi-dimensional policy measure for sustainable food consumption is the recent introduction of national dietary guidelines inspired by the principles of the planetary health diet recommended by the Lancet Commission (42), e.g., in the Nordic countries.

In light of the fact that our study revealed consumer segments with different ‘areas of concern’, the current discussion about multi-dimensional sustainability labels (e.g., ‘Eco-score’ in France) seems highly relevant, yet not without dangers. It seems especially important to stop the silo thinking and merge the dimensions of healthy and nutritious diets, the environment, the climate, and social impacts into comprehensive policy instruments.

### 5.1. Limitations and future research

Cluster analysis is a widely used approach for segmenting individuals based on similarities in their behaviors, however, there are some limitations associated with this method. One of the major drawbacks of cluster analysis is its subjective nature, as the researcher has to make decisions regarding the number of clusters and which clustering technique to use. Additionally, clusters are never fully homogeneous, i.e., there may be individuals in each cluster who do not completely fit into the defined group. Another limitation of cluster analysis is, that it is a descriptive method, and it does not allow to draw conclusions about cause-effect relationships between variables. This means that while cluster analysis can be useful in identifying differences between groups, it cannot explain the causes behind those distinctions. Despite those limitations, cluster analysis was chosen for this study because it facilitated the exploration of the multi-dimensional nature of the phenomenon being studied, which involved three explanatory variables associated with sustainable consumption behavior.

Moreover, using only one indicator to measure healthy food consumption (consumption of sweets, chocolates, and bakery products) is a clear weakness of the study. Moreover, using expenditure value for meat probably hides differences in consumption quantities of different types of meat (e.g., the climate impacts of beef and poultry are very different). Additionally, the indicator for meat consumption did not include data on purchases of processed meat, which is another limitation, e.g., because organic consumers are likely to eat less processed meat (7). However, the additional profiling variables used to characterize the segments purchase patterns suggest that the indicators were successful in measuring sustainable food consumption behavior.

A further constraint of this study is, that the GfK did not provide information on the gender of the survey participants, which is a variable that has been identified as highly pertinent to sustainable consumption. Furthermore, the purchase data was available only at the household level. However, the purchase of sweet snacks is most often an individual choice. Some purchase behaviors (e.g., buying a snack at work) are probably underrepresented in the data.

Future research should further analyze real purchase data and explore whether consumers’ behavior changes regarding different sustainability dimensions and how the attitude-behavior gap develops *over time*. Future research might also investigate how consumers with positive attitudes toward different sustainable production methods (e.g., environmentally friendly, local, animal welfare) could be motivated to transform these attitudes into purchase intentions and finally real purchase behavior. This is specifically interesting for the large segment of ‘mainstream consumers’. Future research should put emphasis on this rather indifferent segment and find ways to nudge people of this cluster to behave more sustainably. Moreover, a further dimension of sustainable food consumption should be added in future studies: food waste behavior. It would be interesting to analyze how the reduction of food waste relates to healthy, environmentally-friendly and climate-friendly food choices.

## Data availability statement

The datasets presented in this article are not readily available because we got the household panel data from the research institute GfK and are not permitted to share the data. Requests to access the datasets should be directed to ischaeufeleelbers@unibz.it.

## Funding

This work was supported by funds of the Federal Ministry of Food and Agriculture (BMEL) based on a decision of the parliament of the Federal Republic of Germany via the Federal Office for Agriculture and Food (BLE) under the Federal Program for organic Farming and Other Forms of Sustainable Agriculture. The project “Analysis of household panel data for the identification of sales potential among customers with low, medium and high organic purchase frequency” was carried out at the University of Kassel, Department of Agricultural and Food Marketing [grant number 2814OE016]. We are grateful to the Free University of Bozen-Bolzano for providing a publishing fund.

## Author contributions

IS-E: methodology, formal analysis, writing–original draft, and visualization. MJ: supervision, conceptualization, and writing–review and editing. All authors contributed to the article and approved the submitted version.

## Conflict of interest

The authors declare that the research was conducted in the absence of any commercial or financial relationships that could be construed as a potential conflict of interest.

## Publisher’s note

All claims expressed in this article are solely those of the authors and do not necessarily represent those of their affiliated organizations, or those of the publisher, the editors and the reviewers. Any product that may be evaluated in this article, or claim that may be made by its manufacturer, is not guaranteed or endorsed by the publisher.
