# Supplementary material for: Consumer segmentation based on three dimensions of sustainable food consumption: a simultaneous analysis of meat, organic food, and sweet snack purchases based on household panel data in Germany
Source: Front Nutr. 2023 Jun 27;10:1140636. doi: 10.3389/fnut.2023.1140636 (PMC10335000; doi:10.3389/fnut.2023.1140636)
Supplement: Supplementary file 1 [file Data_Sheet_1.docx]

1. Tables in the Appendix

Table A.1: Food-values scales

| Scale | Item | Cronbach’s alpha |
| --- | --- | --- |
| Body-consciousness | I take care of my figure while eating and drinking | 0.814 |
|  | I take great care to eat as little fat as possible |  |
|  | I would describe myself as very conscious about my figure |  |
|  | I make sure that I do not exceed a certain number of calories per day to stay slim |  |
|  | I pay strict attention to the sugar content of food |  |
|  | I pay strict attention to a low carbohydrate diet |  |
|  | I take care to eat only light, healthy food in the evening |  |
| Convenience food | I only buy fresh products instead of e.g., canned food or frozen food (reversed) | 0.772 |
|  | For food preparation I prefer fresh ingredients (reversed) |  |
|  | I can hardly image cooking without convenience products (like instant gravy, frozen food, canned food). |  |
|  | Nowadays, canned food tastes as good as fresh food to me |  |
|  | Convenience products from the refrigerated section are as good as homemade |  |
|  | Ready-made refrigerated meals are as good as self-made meals |  |
| Environmental protection | In my household, I can do little to protect the environment (reversed) | 0.743 |
|  | Nowadays, too much fuss is made about the environment (reversed) |  |
|  | What is currently done to protect the environment is absolutely sufficient (reversed) |  |
|  | I care little about the environmental impact of products (reversed) |  |
|  | I obtain information about which foods are environmentally harmful and stop buying them |  |
|  | I am willing to spend more money for environmentally-friendly packaging |  |
|  | For environmental protection, one must personally accept even substantial restrictions |  |
|  | I consciously buy more products that are less harmful to the environment |  |
| Fair trade | I deliberately buy products from "Fair Trade" (e.g., FAIRTRADE) | 0.812 |
|  | I am willing to spend more money for fair-trade products |  |
|  | For me, fair treatment of the producers in the country of origin is also part of sustainability |  |
| Fast food | At home we cook hot meals every day (reversed) | 0.765 |
|  | I often eat in a fast-food restaurant or at a snack bar |  |
|  | If I do not feel like preparing food, I order from the delivery service (e.g., pizza service) |  |
|  | I often have breakfast on the way to work during the week, e.g., in a fast-food restaurant or in a bakery |  |
|  | Due to lack of time, I hardly cook anymore |  |
|  | Today I eat less often at home and more often on the road than a few years ago |  |
| Avoiding health risks | In my household I pay great attention to gentle, low-irritant food | 0.776 |
|  | I pay attention to what I eat and drink, because I have to take care of my health |  |
|  | In my diet I avoid everything that is harmful to my health |  |
|  | With the purchase of food, the topic Cholesterol plays a role |  |
|  | I take great care to eat with as little salt as possible |  |
|  | I take great care to avoid foods containing caffeine |  |
|  | To prevent digestive problems, I use special food |  |
| Local food | I do not care if my food is from Germany or any other country (reversed) | 0.831 |
|  | For me, food from Germany is the best quality |  |
|  | When I have the choice, I definitely buy food from Germany |  |
|  | When I have the choice, I prefer local food |  |
|  | I am willing to pay more for local products |  |
| Natural food | I dislike products containing preservatives | 0.756 |
|  | When shopping for food, I am careful to choose products without any additives |  |
|  | I dislike products containing flavor enhancers (e.g., glutamate) |  |
|  | I would never buy genetically engineered food |  |
| Quality and enjoyment | I can really get enthusiastic about specialties from other countries | 0.773 |
|  | I treat myself to delicacies once in a while. |  |
|  | I like cooking extravagant dishes |  |
|  | I like treating myself to fine food |  |
|  | I enjoy trying out foreign specialties |  |
|  | I demand high standards when it comes to food and drinks |  |
| Simple and easy cooking | I take a lot of time for cooking (reversed) | 0.737 |
|  | I prefer to cook dishes quickly |  |
|  | The easier to cook, the better |  |

Table A.2: Demographic data of segments

|  | medium organic buyers | heavy organic buyers | heavy meat buyers | sweet snack enthusiasts | mainstream | total |
| --- | --- | --- | --- | --- | --- | --- |
| Weighted per capita net income (in %)^1^ | | | | | | |
| Up to 749 € | 4.9^a^ | 5.3^a,c^ | 11.9^b^ | 11.9^b^ | 10.0^c^ | **10.4** |
| 750–999 € | 6.9^a^ | 4.5^a^ | 12.0^b^ | 14.2^c^ | 11.1^b^ | **11.5** |
| 1000–1249 € | 14.3^a^ | 16.7^a,b^ | 18.8^b^ | 19.5^b^ | 17.5^b^ | **18.0** |
| 1250–1499 € | 17.6^a,b^ | 13.6^b^ | 19.9^a,b^ | 18.8^a,b^ | 20.7^a^ | **19.8** |
| 1500–1999 € | 25.0^a^ | 18.2^a,b^ | 21.1^b^ | 19.6^b^ | 21.3^b^ | **21.2** |
| 2000 € and more | 31.2^a^ | 41.7^c^ | 16.3^b^ | 16.0^b^ | 19.3^d^ | **19.2** |
|  | **100.0%** | **100.0%** | **100.0%** | **100.0%** | **100.0%** | **100.0%** |
| Education of the head of calendar (in %) | | | | | | |
| Secondary general school | 13.2^a^ | 10.6^a^ | 27.0^b^ | 23.1^c^ | 21.8^c^ | **22.5** |
| Intermediate Secondary School | 25.0^a^ | 18.9^a^ | 33.3^b,c^ | 36.0^b^ | 33.1^c^ | **32.9** |
| Special upper secondary school | 10.2^a^ | 11.4^a,b^ | 7.3^b^ | 7.7^b^ | 8.0^a,b^ | **8.0** |
| Grammar school | 15.9^a^ | 11.4^a^ | 14.1^a^ | 14.0^a^ | 14.0^a^ | **14.1** |
| University | 35.6^a^ | 47.7^c^ | 18.3^b^ | 19.2^b^ | 23.1^d^ | **22.5** |
|  | **100.0%** | **100.0%** | **100.0%** | **100.0%** | **100.0%** | **100.0%** |
| Age of the head of household (in %) | | | | | | |
| up to 29 years | 2.2^a^ | 3.0^a^ | 1.0^b^ | 1.7^b^ | 2.5^b^ | **1.9** |
| 30-39 years | 11.8^a^ | 15.2^a^ | 5.9^b^ | 11.5^a^ | 11.3^a^ | **10.1** |
| 40-49 years | 15.4^a^ | 15.9^a,b^ | 16.2^a^ | 20.8^b^ | 16.4^a^ | **17.2** |
| 50-59 years | 22.4^a^ | 29.5^a,b^ | 30.3^b^ | 23.5^a^ | 22.7^a^ | **24.8** |
| 60-69 years | 26.3^a^ | 18.2^b^ | 27.6^a^ | 21.4^b^ | 21.5^b^ | **23.3** |
| 70 years and more | 21.9^a^ | 18.2^a,b^ | 19.0^a^ | 21.1^a^ | 25.5^b^ | **22.6** |
|  | **100.0%** | **100.0%** | **100.0%** | **100.0%** | **100.0%** | **100.0%** |
| Household size (average number of people living in the household) | | | | | | |
|  | 2.05 | 2.11 | 2.21 | 2.07 | 2.09 | 2.11 |

a,b,c,d shares of segments with different letters differ significantly (p<0.05). ^1)^ First Adult + 0.7*following adult + 0.5*children
